# Giant Cell Tumor of the Tendon Sheath of the Thumb: A Case Report

**DOI:** 10.7759/cureus.48162

**Published:** 2023-11-02

**Authors:** Rayed Qamar, Faiz Ahmad, Madhav Chowdhry, Mit Parikh, Kavya Joshi

**Affiliations:** 1 Orthopaedic Surgery, Geetanjali Medical College and Hospital, Udaipur, IND; 2 Orthopaedic Surgery, Indraprastha Apollo Hospitals, New Delhi, IND; 3 Orthopaedic Surgery, Jawaharlal Nehru Medical College, Aligarh Muslim University, Aligarh, IND; 4 Radiodiagnosis, Geetanjali Medical College and Hospital, Udaipur, IND

**Keywords:** tumor, benign, excision, hand, gct tendon sheath

## Abstract

The giant cell tumor of the tendon sheath (GCTTS) is a benign nodular tumor that is found on the tendon sheath of hands and feet. It is the second most common tumor of the hand, next only to ganglion cysts. Several hypotheses were formulated about the etiological factors of these tumors, but still, there is not a common opinion on etiology, prognostic factors, and recurrence rate. We report a case of GCTTS in a young male where a lesion was identified in his left thumb. Although marginal excision is the treatment of choice, it is often difficult to perform due to the location and the strict adherence of the tumor to the tendon or neurovascular bundles. The primary issue with the treatment lies in its elevated recurrence rates. Apart from cases of incomplete excision, there is a lack of consensus regarding the impact of other risk factors on the likelihood of recurrence.

## Introduction

Giant cell tumor of the tendon sheath (GCTTS) commonly appears as a slowly developing soft tissue lump that gradually grows over months to years. It is referred to by various other names, including pigmented villonodular tenosynovitis, fibrous xanthoma, xanthogranuloma, and localized nodular synovitis [1]. These multiple names stem from the lack of clarity regarding its specific pathological characteristics [1].

GCTTS is characterized by abnormal synovial tissue growth [2]. Etiological factors encompass trauma, inflammation, metabolic disorders, and a potential neoplastic origin [1]. According to the most widely accepted theory, it is a form of hyperplasia that occurs as a reaction or regeneration in response to an inflammatory process [2]. GCTTS is the second most prevalent tumor in the hand [3]. GCTTS can be categorized into two forms: (i) the localized form, which is generally benign and primarily affects the hand and fingers, and (ii) the diffuse form, which is more aggressive and typically develops in larger joints [4]. The typical location of this tumor is usually the distal interphalangeal joint of the long and ring fingers [5]. The chances of recurrence are higher when the degenerative joint disease is present near the distal interphalangeal joint of the finger or interphalangeal joint of the thumb, along with radiographic signs of osseous pressure erosion [2].

## Case presentation

A 31-year-old man who was a manual laborer working frequently with heavy vibrating machinery presented with complaints of pain and swelling on the distal part of the left thumb for the past three months. On physical examination, a hard tender swelling of 10x5 mm was found on the volar aspect of the thumb. However, there was no numbness or loss of grip strength and no color or texture changes in the skin of the thumb. Ultrasonography was suggestive of a round hypoechoic nodule with irregular margins measuring 9.7x6.3x6 mm seen in pulp overlying the distal phalanx of the left thumb; however, in view of occupational history, a possibility of inflammatory pseudotumor was suggested.

**Figure 1:**
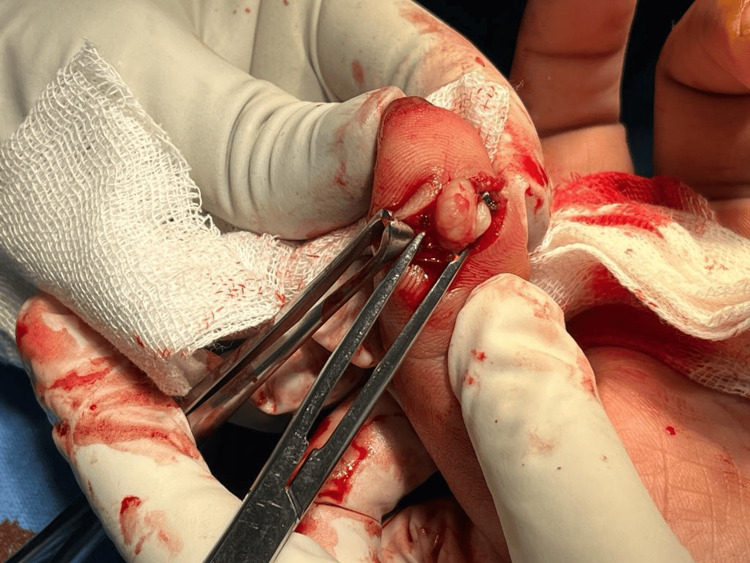
Intraoperative photograph of tumor

**Figure 2:**
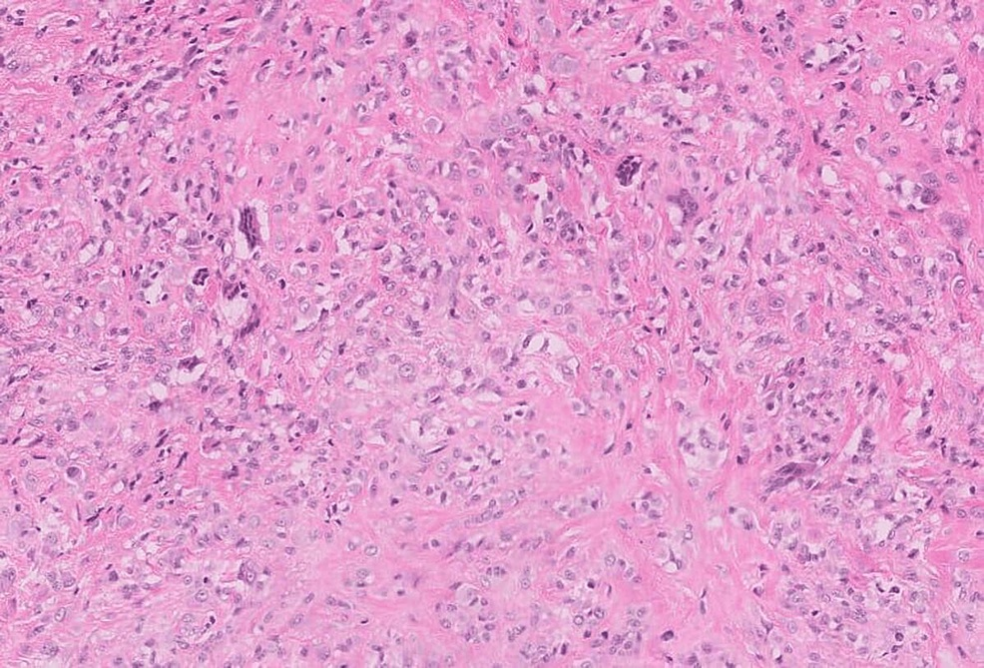
Foamy macrophages and inflammatory cells

**Figure 3:**
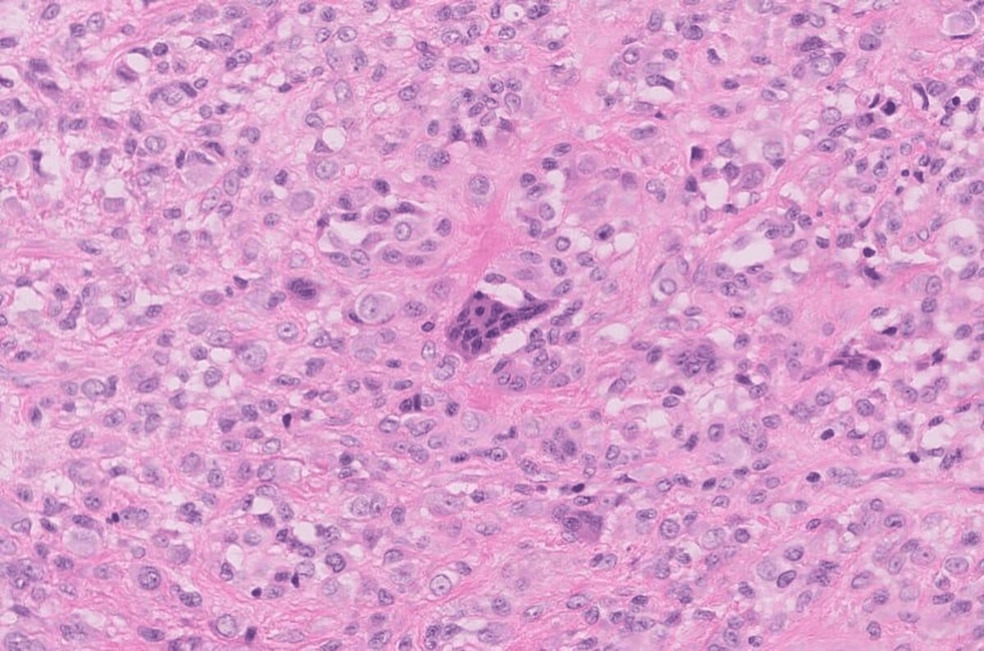
Multinucleated giant cells in background of few epitheloid cells

The patient was planned for surgery and a marginal excision of the tumor was done by volar midline oblique approach. A tumor of size approximately 1x0.5 cm was excised (Figure 1). The resected tumor with margins was sent for histopathological examination (HPE). HPE revealed nodular architecture composed of abundant mononuclear cells, with occasional foamy macrophages and sparse inflammatory cells (Figure 2). Few epithelioid cells along with interspersed multinucleated giant cells were seen favoring giant cell tumors (Figure 3). The histopathological findings indicated that these masses were in line with GCTTS, showing no signs of malignancy.

The patient was discharged on the third postoperative day. There was follow-up every three months for a period of two years, and no signs or symptoms of recurrence were observed. The patient conveyed his gratitude for the absence of any potential deformity or numbness discussed during preoperative communication. The patient achieved a complete recovery with unrestricted movement and no swelling. There were no signs of recurrence in both clinical and radiological examinations.

## Discussion

Despite the extensive body of published literature on hand GCTTS, the treatment continues to pose a significant challenge for hand surgeons [6]. Given the tumor's potential to infiltrate joints and bone cortices, as well as extend into tendon sheaths and encase neurovascular structures, striking the right balance between thorough and aggressive tumor removal while preserving essential tissues presents significant challenges [6].

While the exact cause of GCTTS is not well-defined, many researchers propose the hypothesis that it arises from a reactive inflammatory process [7]. Hand tumors can be differentially diagnosed with conditions such as lipomas, hemangiomas, foreign bodies, myxoid cysts, synovial carcinomas, tophaceous gout, glomus tumors, tuberous osteitis, epidermal cysts, fibromas, and metastases [7]. Complete local excision is the treatment of choice [8]. Definitive diagnosis can be made only by HPE. Only 20-30% of GCTTS are diagnosed before surgery [9], highlighting the importance of HPE. Although the condition commonly follows a benign course, it is noteworthy that there have been isolated reports of malignant transformation in exceptional cases [10]. The diffuse type has the potential to exhibit local aggressiveness, and there have been reports of potential multiple recurrences and the risk of malignant transformation. Choughri et al. suggested that the postsurgical tumor recurrence rate ranged approximately between 15% and 45% [11]. According to Williams et al., the comprehensive rates of recurrence varied between 7% and 44% [12]. Hakan documented a recurrence rate of 6% [6]. Currently, an essential focus is on radical surgical intervention to minimize the likelihood of recurrence [13].

## Conclusions

While GCTTS is not frequently discussed among orthopaedics, it is crucial to acknowledge their presence. This is due to its uncertain origins and behavior, which can lead to it resembling other medical conditions. The case reported serves as a reminder to consider a variety of diagnoses when evaluating hand tumors and to pursue HPE and excisional treatment when deemed necessary.
